# On the Worrying Fate of Data Deficient Amphibians

**DOI:** 10.1371/journal.pone.0125055

**Published:** 2015-05-12

**Authors:** Javier Nori, Rafael Loyola

**Affiliations:** 1 Centro de Zoología Aplicada, Facultad de Ciencias Exactas Físicas y Naturales, Universidad Nacional de Córdoba and Instituto de Diversidad y Ecología Animal—Consejo Nacional de Investigaciones Científicas y Técnicas and Universidad Nacional de Córdoba, Córdoba, Córdoba, X5000AVP, Argentina; 2 Laboratório de Biogeografia da Conservação, Departmento de Ecologia, Universidade Federal de Goiás, CP 131, CEP 74001–970, Goiânia, Goiás, Brazil; University of Brasilia, BRAZIL

## Abstract

The ‘Data Deficient’ (DD) category of the *IUCN Red List* assembles species that cannot be placed in another category due to insufficient information. This process generates uncertainty about whether these species are safe or actually in danger. Here, we give a global overview on the current situation of DD amphibian species (almost a quarter of living amphibians) considering land-use change through habitat modification, the degree of protection of each species and the socio-political context of each country harboring DD species. We found that DD amphibians have, on average, 81% of their ranges totally outside protected areas. Worryingly, more than half of DD species have less than 1% of their distribution represented in protected areas. Furthermore, the percentage of overlap between species’ range and human-modified landscapes is high, at approximately 58%. Many countries harboring a large number of DD species show a worrying socio-political trend illustrated by substantial, recent incremental increases in the *Human Development Index* and lower incremental increases in the establishment of protected areas. Most of these are African countries, which are located mainly in the central and southern regions of the continent. Other countries with similar socio-political trends are in southeastern Asia, Central America, and in the northern region of South America. This situation is concerning, but it also creates a huge opportunity for considering DD amphibians in future conservation assessments, planning, and policy at different levels of government administration.

## Introduction

The fact that amphibians are undergoing a global crisis is not new [1–3]. Whereas more than 41% of the living amphibians species are currently threatened [4,5], almost a quarter of all species are classified as Data Deficient (DD) according to the International Union for the Conservation of Nature (IUCN). This category is assigned to those species for which data is insufficient to assess extinction risk [6]. Eclipsed by those species flagged with a threat status, DD species often remain marginalized in most conservation planning and policies [7–9]. However, the degree of threat they face may vary considerably [10,11]. Hence, considering all different DD species as equivalents during conservation planning and policy might lead to the loss of species before they can be precisely placed into a formal threat category.

In recent years, some authors have called attention to this issue [9–13]. Trindade-Filho et al. [9] showed that priority sites for amphibian conservation change considerably when DD species are included in spatial prioritization analyses. Butchart & Bird [12] and Morais et al. [10] attempted to address the current threat status of different DD species. Recently, Howard & Bickgford [11] predicted the extinction risk of DD amphibians at the global scale. These authors showed that DD amphibian species are likely to be more threatened with extinction than the other amphibian species which were fully assessed by the IUCN.

The situation of DD amphibians is now critical and our aim is to provide additional evidence to address this urgent problem. Here, we give a global overview on the current situation of DD amphibians, integrating into our assessment: (1) land-use change through habitat modification, which is the most threatening human pressure upon amphibians; (2) the degree of protection of each species; and (3) the socio-political trend of each country harboring DD species in the world. Although we do not want to assess the ‘real’ threat status of DD species, our approach is a useful perspective of the problem, in a world where conservation and social policy should go hand in hand.

## Materials and Methods

We obtained shape files of terrestrial protected areas around the globe from the World Database of Protected Areas website [14]. We selected only those protected areas with the ‘designated’ status (i.e., we did not consider ‘inscribed’, ‘non-reported’, nor ‘proposed’ protected areas) from all six management categories defined by the IUCN (I to VI), totaling 126,280 protected areas.

For the amphibian dataset, we downloaded available digital range maps for 6316 species in the IUCN database [15]. This dataset represents 89.5% of all amphibian extant species according to Frost [16]. Using spatial analysis, we associated species’ conservation status [15] to their geographic distribution and filtered our database by DD amphibian species ending up with1545 species (which corresponds to 24% of all amphibians). Although the global scale of our analyses implies the need to assume some level of commission and omission errors in species distribution when using range maps, these maps accurately represent the known distribution of most of the species included being useful and appropriate for global analyses [17].

To determine how much area of each species geographic distribution overlaps with protected areas, we first calculated the surface of distribution for each amphibian species. Then, using ‘spatial analysis’ tools in *ArcGIS 10*.*2*2, we calculated the surface of each species' range overlapping with protected areas. With this information we calculated the proportion of protected ranges in relation to the total extent of the distribution of each species. Finally, we grouped species into different ‘categories of protection’ and drew a histogram of frequencies.

To estimate the percentage of the distribution of each species overlapping human-modified areas, we obtained information on human impact on natural environments from the *Anthropogenic Biomes of the World (v*. *1)* [18] and we reclassified the original files (with 21 categories of Anthropogenic Biomes) into two different categories: (a) wild areas and (b) human-dominated landscapes (for details please see Brum et al. [19]). Then, using the *GIS* platform, we superimposed our binary raster of Anthropogenic Biomes with range maps of each DD amphibian species and determined the percentage of each species’ range overlapping with wild areas or any of the other categories of human-dominated landscapes. Considering the centroid of the geographic range of each DD species (also calculated in *ArcGIS 10*.*2*2), we inferred the continent in in which each species occur (subcontinent in the case of America) and grouped all our results by continent.

For each country of the world, we downloaded information from the *Human Development Index* (HDI) between 2000 and 2012 from the Human Development Indicators web page (http://hdr.undp.org), and calculated the variation of the index in this period. Then, using the ‘join data’ tool of *ArcGis 10*.*2*, we included this information in a vector file of the political countries of the world, generating a map showing, for each county, the recent variation in HDI (see S1 Fig). Additionally, from the attribute table of the vector file with designated protected areas, we selected all protected areas established between 2000 and 2012 and created a new vector file with this information. After that, we merged this file with the vector file of the countries and calculated the surface of protected areas established in every country in the same period. Finally, using the surface of protected areas in each country and each country’s area, we calculated the recent (2000–2012) increment in in protected area surface per unit area for each country (see S2 Fig).

We combined the map showing the recent variation in HDI with the map showing the increment in protected area surface for the same period to generate a bivariate map (showing both variables together). This map reflects the sociopolitical trend of each country. To generate our final map, we combined this sociopolitical context map with the centroid of the distribution of each DD species, discriminating between gap species (i.e., those species having a proportion of their geographic distribution protected in less than 1%) and all other species. Therefore, along with DD species’ distribution, we could discriminate (for each country), recent human development trends (which are also strongly related to Gross Domestic Product per capita, see www.bit.ly/UJa7mP and www.gapminder.org/news/hdi-surprisingly-similar-to-gdpcapita) and the recent allocation of resources to the establishment of protected areas.

## Results

**Fig 1:**
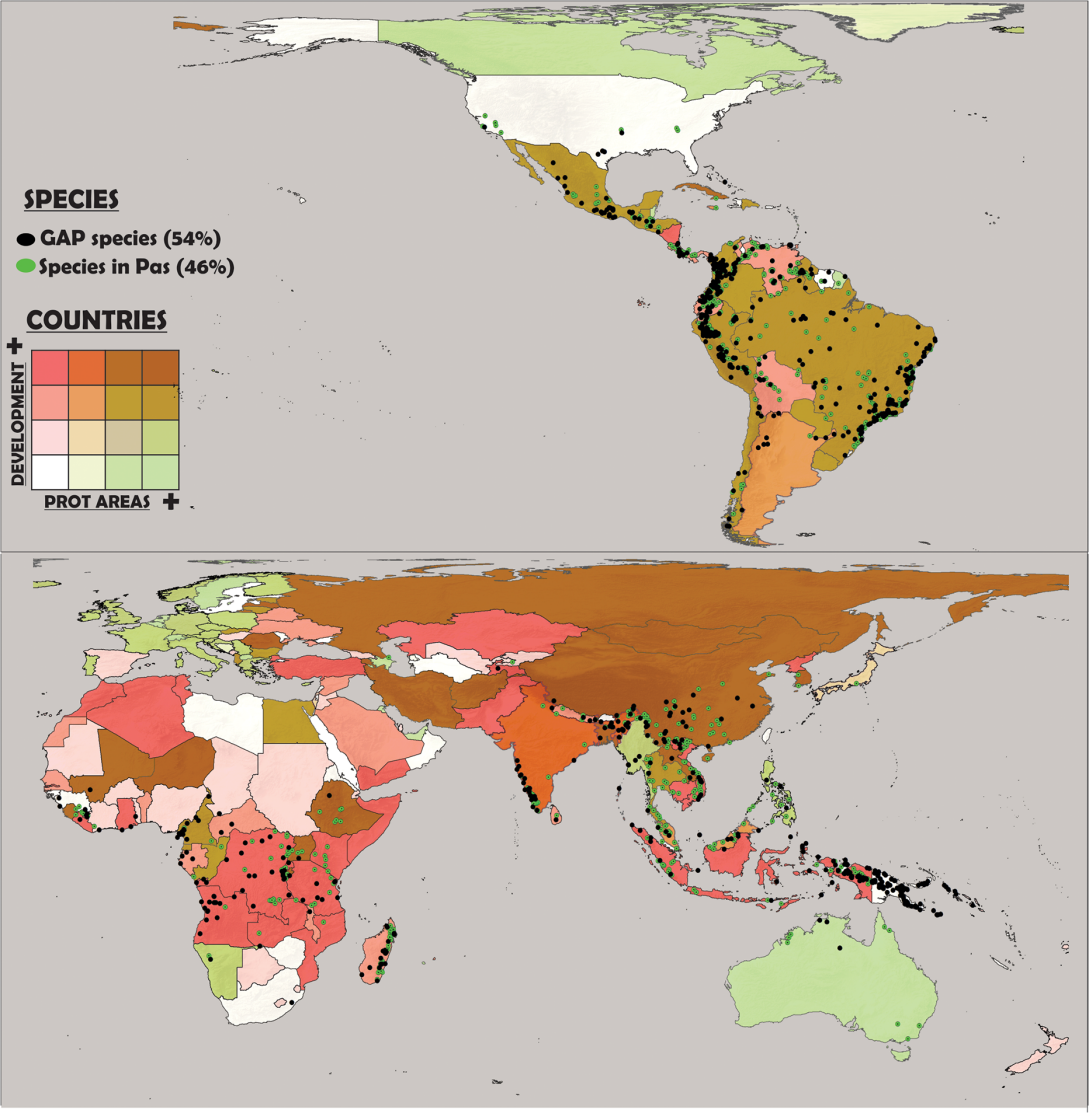
The gap in the protection of Data Deficient species in relation to current socio-political trends in different countries. Map showing (a) the geographic centroid and degree of protection (showed in two categories: gap species and those found in protected areas) for Data Deficient amphibian species; and (b) variation in Human Development Index and protected area surface in each country of the world, from 2002 to 2012.

**Fig 2:**
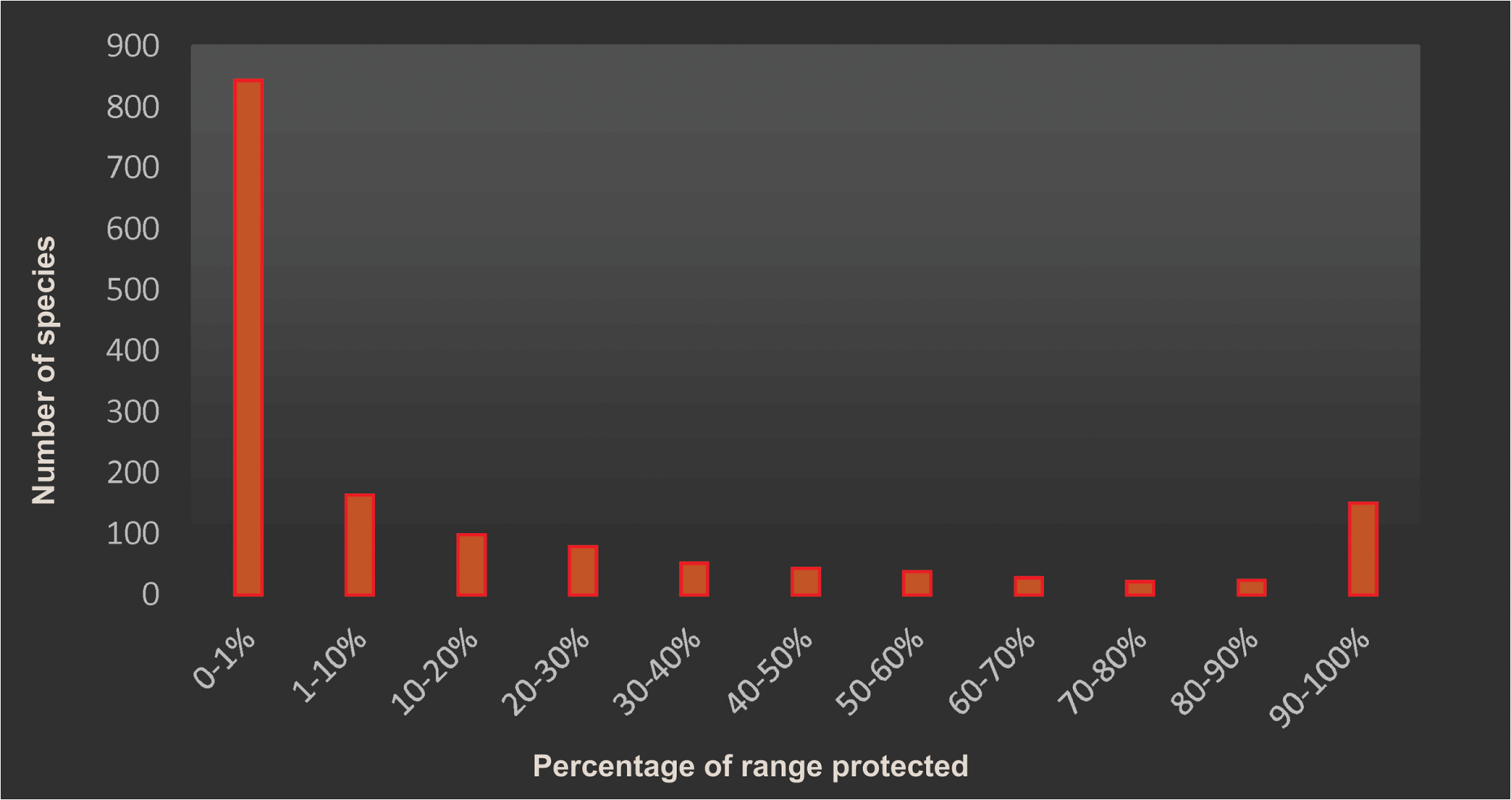
Current level of protection for Data Deficient amphibian species of the world. Histogram showing the average percentage of geographic distribution of Data Deficient amphibian species found inside protected areas in the world.

Data Deficient amphibians have, on average, 81% of their geographic distribution completely outside of protected areas (although such overlap is highly variable between continents). While in Oceania DD species have (on average) less than 3% of their distribution currently protected, in Latin America and Asia they have more than 20% of their distribution overlapping with protected areas (Table 1, Fig 1). More than half of DD species (54%) have less than 1% of their distribution overlapping protected areas and 10% of species are protected in surfaces of between 1% and 10% of their distribution. In contrast, another 10% of species are totally protected by currently established protected areas (Fig 2). The percentage of overlap between species’ geographic distribution and human-modified landscapes was high (mean of 58%), but it was also highly variable between continents. For example, in Oceania DD species had (on average) 22% of their distribution overlapping human-dominated landscapes whereas, in Africa more than 75% of the DD species range overlapped with human-dominated landscapes (Table 1, Fig 1).

**Table 1:** Number of Data Deficient amphibian species, mean percentage of overlap of their geographic distribution with human-dominated landcapes, and mean percentage of their geographic distribution protected in different continents.

Continent	Number of Data Deficient species	% of overlap with human-dominated landscapes	% of geographic distribution protected
Africa	202	75.57	16.13
Latin America	801	54.47	21.32
North America	13	49.16	18.41
Asia	410	66.99	23.40
Europe	1	93.33	0.00
Oceania	118	22.41	2.72

Many countries around the world showed worrying socio-political trends for the conservation of DD species. This context is illustrated by a recent, large-scale incremental growth in HDI and lower incremental growth in the establishment of protected areas, most of which are in: Africa (mainly in the central and southern regions), Southeastern Asia, Central America, and the northern region of South America. In contrast, a best-case scenario for protection (i.e., low recent incremental growth in HDI because of previous high incremental growth and large incremental growth in protected surfaces) was observed in Europe, Canada, and Australia (Fig 1).

## Discussion

Like other authors [9–11], we must emphasize that the fate of DD amphibians is extremely uncertain and the these species require urgent attention. Our results provide valuable information to reinforce that these species are actually facing a higher pressure, which will eventually increase their extinction risk leading to their premature ruin. Further, we link for the first time this pattern with current socio political trends, species’ level of protection, and the habitat modification found in regions inhabited by DD species.

At the global scale, species’ level of protection is minimal, the percentage of overlap between their geographic ranges and human-dominated landscapes is extremely high, and the socio-political trends found in many countries harboring a high number of these species is not encouraging. In fact, quite the opposite, this observed context complicates the picture for DD amphibians. Further complicating the situation, many DD species occur in countries displaying worst-case scenarios, with rapid economic growth and no precise environmental policies.

Although resources diverted into conservation actions are limited, and so prioritizing conservation goals is absolutely necessary, this prioritization must be made on the basis of all available information. Hence, it is very important to note that in most cases, recommendation for conservation intervention and conservation policies give strong importance (or make strong distinction) to species currently assigned into IUCN threat categories, without considering DD species, or at least considering them equal that species in low threat [7,8,20,21]. Our decision and policy makers are doing so even when we do know that many DD species are actually threatened [10,11], and that their inclusion into conservation systematic plans could produce major changes [9]. These decisions are generating “strongly unbalanced recommendations” regarding DD species. Therefore, we believe that DD amphibians must receive special attention in conservation planning, and our results could provide valuable information to be used in this sense.

Here we identified regions such as the Atlantic Forest, the Tropical Andes, Southeastern Asia, Central and South Africa, and Madagascar in which this particular issue with DD species has become extremely critical. Most of these regions have been identified as key regions for biodiversity conservation [4,8,22] and as regions with great gaps in their network of protected areas [20]. Recently, the same regions have been further identified as ‘regions of high risk’ for DD amphibians (considering various criteria, including: life-history traits, environmental classification, and lack of knowledge) [11]. Our results reinforce this idea and, more importantly, shows the socio-political context for each of these world regions, which makes the problem even more pressing.

Latin America ranks first in terms of the magnitude of this problem. Fortunately, social development trends and conservation policy in many countries that DD species inhabit (e.g., Brazil, Peru, Colombia, and Mexico) are not as threatening given that these countries have also shown important incremental growth in their protected surfaces over the last years, along with important incremental growth in HDI, in most cases [14,23]. Other countries in the Western Hemisphere, such as Panama, Nicaragua, and Bolivia, which hold high numbers of DD amphibians, show a troublesome reality displayed by a lack of conservation policies in a context of increased human development.

Southeastern Asia as well as a great portion of Africa and Madagascar are also critical regions as they hold a high number of DD amphibian species and have a sociopolitical context very discouraging for conservation actions. Habitat loss and over-exploitation of natural resources have previously been identified as major causes of amphibian decline in these regions [1]. If the economic growth of poor and developing countries is mostly based on the exploitation of natural resources (increasing the economy and social development of nations), this will represent a somber and dystopian future for DD species. In this sense, when decision and policy makers of these countries are ready to propose conservation actions it would be important that these actions are not biased and give strong consideration to DD species.

Although almost a quarter of living amphibians are classified as Data Deficient, we believe that this is an excellent time to act. The targets are clear, and the number and size of protected areas should significantly increase in the following years, especially toward more "areas for particular importance for biodiversity" [24]. Hopefully, by 2020, decision makers and the scientific community have stopped turning a blind eye to DD species of amphibians, considering and properly including them in conservation systematic plans, so that this urgent problem is addressed and realistic solutions are fulfilled.

## Conclusion

As other authors have previously illustrated, here we showed that most Data Deficient amphibian species are facing major human pressures. Even worse, policies and recommendations are in overall unbalanced, leaving out, or giving minor importance to those species, making their fate extremely uncertain. The socio-political context of countries harboring many of these species—especially in central Africa and Madagascar, Southeastern Asia, and Central America—suggest that the problem is even more urgent in these places. For these reasons, give special attention to DD species in conservation planning (especially in these countries mentioned) is an absolute need.

## Supporting Information

S1 Fig Variation in the Human Development Index between 2000 and 2012 on each country. (TIF)

S2 Fig Variation in the surface of protected areas between 2000 and 2012 on each country (Km^2^ of protected surface between 2000 and 2012 / Million of Km^2^). (TIF)
